# Detailing the effects of polypharmacy in psychiatry: longitudinal study of 320 patients hospitalized for depression or schizophrenia

**DOI:** 10.1007/s00406-021-01358-5

**Published:** 2021-11-25

**Authors:** H. H. Stassen, S. Bachmann, R. Bridler, K. Cattapan, D. Herzig, A. Schneeberger, E. Seifritz

**Affiliations:** 1 Institute for Response-Genetics, Department of Psychiatry, Psychotherapy and Psychosomatics, Psychiatric University Hospital, CH-8032 Zurich, Switzerland; 2 Department of Psychiatry, Psychotherapy, and Psychosomatics, University of Halle, D-06112 Halle, Germany; 3 Psychiatric Hospital, Clienia AG, CH-9573 Littenheid, Switzerland; 4 Sanatorium Kilchberg, CH-8802 Kilchberg, Switzerland; 5 University Hospital of Psychiatry and Psychotherapy, University of Bern, Bern, Switzerland; 6 Department of Psychiatry, Psychotherapy and Psychosomatics, Psychiatric University Hospital, CH-8032 Zurich, Switzerland

**Keywords:** Polypharmacy, Monotherapy, Antidepressants, Antipsychotics, Efficacy, Side effect profiles, Concurrent medications

## Abstract

Current treatment standards in psychiatry are oriented towards polypharmacy, that is, patients receive combinations of several antidepressants, antipsychotics, mood stabilizers, anxiolytics, hypnotics, antihistamines, and anticholinergics, along with other somatic treatments. In tandem with the beneficial effects of psychopharmacological drug treatment, patients experience significant adverse reactions which appear to have become more frequent and more severe with the rise of ubiquitous polypharmacy. In this study, we aimed to assess today’s acute inpatient treatment of depressive and schizophrenic disorders with focus on therapeutic strategies, medications, adverse side effects, time course of recovery, and efficacy of treatments. Of particular interest was the weighing of the benefits and drawbacks of polypharmacy regimens. We recruited a total of 320 patients hospitalized at three residential mental health treatment centers with a diagnosis of either schizophrenic (ICD-10: “F2x.x”; *n* = 94; “F2 patients”) or depressive disorders (ICD-10: “F3x.x”; *n* = 226; “F3 patients”). The study protocol included (1) assessment of previous history by means of the SADS Syndrome Check List SSCL-16 (lifetime version); (2) repeated measurements over 5 weeks assessing the time course of improvement by the Hamilton Depression Scale HAM-D and the Positive and Negative Syndrome Scale PANSS, along with medications and adverse side effects through the Medication and Side Effects Inventory MEDIS; and (3) the collection of blood samples from which DNA and serum were extracted. Polypharmacy was by far the most common treatment regimen (85%) in this study. On average, patients received 4.50 ± 2.68 medications, consisting of 3.30 ± 1.84 psychotropic drugs, plus 0.79 ± 1.13 medications that alleviate adverse side effects, plus 0.41 ± 0.89 other somatic medications. The treating psychiatrists appeared to be the main determining factor in this context, while «previous history» and «severity at baseline» played a minor role, if at all. Adverse drug reactions were found to be an inherent component of polypharmacy and tended to have a 2–3 times higher incidence compared to monotherapy. Severe adverse reactions could not be attributed to a particular drug or drug combination. Rather, the empirical data suggested that severe side effects can be triggered by virtually all combinations of drugs, provided patients have a respective vulnerability. In terms of efficacy, there were no advantages of polypharmacy over monotherapy. The results of this study underlined the fact that polypharmacy regimens are not equally suited for every patient. Specifically, such regimens appeared to have a negative impact on treatment outcome and to obfuscate the “natural” time course of recovery through a multitude of interfering factors. Evidence clearly speaks against starting just every therapeutic intervention in psychiatry with a combination of psychopharmaceuticals. We think that it is time for psychiatry to reconsider its treatment strategies, which are far too one-sidedly fixated on psychopharmacology and pay far too little attention to alternative approaches, especially in mild cases where psychotherapy without concurrent medication should still be an option. Also, regular exercises and sports can definitely be an effective therapeutic means in a considerable number of cases. General practitioners (GPs) are particularly in demand here.

## Background

Over the past two decades, stress-induced mental health problems such as psychosomatic disturbances, burn-out conditions, social anxiety, or depressive and schizophrenic disorders were on the rise globally, thus significantly contributing to the burden of disability and mortality, while reducing quality of life. Worldwide, mental health problems account for 21.2% of years lived with disability [1]. Available treatments, though effective, are incomplete since all treatment options are non-causal, so that, for example, antidepressants and antipsychotics that differ greatly in their biochemical design and primary site of pharmacological action display virtually the same insufficient efficacy [2]. Likewise, it is not possible to reliably predict whether a particular patient will respond to a particular treatment or experience certain adverse side effects. And worst of all, there is no long-term cure for a substantial proportion of patients: for example, for 50–60% of patients with schizophrenic disorders (e.g., [3]), and for 35–50% of patients with major depression (e.g., [4, 5]). A solution to this unsatisfactory situation is not to be expected in the near future.

Current treatment standards in psychiatry are oriented towards polypharmacy, that is, patients are no longer treated with one single medication but receive combinations of several antidepressants, antipsychotics, mood stabilizers, anxiolytics, hypnotics, antihistamines, and anticholinergics, along with other somatic treatments. Psychotherapy without parallel medication is not even considered in the vast majority of cases [6]. In parallel with the general acceptance and spread of polypharmacy, the percentage of treatment responders has declined dramatically. About 15 years ago, we found in a cross-comparison of five antidepressants (*n* = 2245) responder rates between 47.5% and 60.9% under monotherapy [2], while the responder rates under antipsychotics lay in the range of some 40% [7]. By contrast, in a naturalistic pilot study of 296 inpatients and 363 outpatients, the patients received an average of 4.6 ± 2.0 concurrent medications and showed response rates of around 35% for major depression and of around 25% for schizophrenic disorders [8]. This meant a general drop of 40% compared to what one observed 20 years ago. The question of declining treatment effects in schizophrenia drug trials has recently been addressed already by several authors [9, 10].

In tandem with the potentially beneficial effects of psychopharmacological drug treatment, patients experience significant adverse side effects which appear to have become more frequent and more severe with the rise of ubiquitous polypharmacy. This became evident, for example, in our recent study on the role of inflammatory processes in depression and schizophrenia, where 85.7% of patients treated for major depression reported adverse side effects (31.0% in severe form), and 81.7% of patients treated for schizophrenic disorders (33.1% in severe form) [6]. Undoubtedly, in a considerable number of cases the beneficial effects of multiple psychopharmacological medications do not outweigh the associated risk of adverse side effects.

There is little to no empirical evidence that demonstrates the advantages of polypharmacy approaches over monotherapy. The most consistent finding comes from a comprehensive Cochrane study, suggesting that a certain subgroup of F2 patients can apparently benefit from antipsychotic polypharmacy without major negative consequences [11]. But here too we have a number of open questions regarding efficacy and long-term safety, and of how to identify the respective patients.

In this observational study, we aimed to assess today’s acute inpatient treatment of major depressive and schizophrenic disorders with focus on therapeutic strategies, medications, adverse side effects, time course of recovery, and efficacy of treatments. Of particular interest was the weighing of the benefits and drawbacks of the polypharmacy approach. Specifically, our study addressed the following questions: (1) What is the prevalence of polypharmacy in three typical psychiatric hospitals (residential mental health treatment centers)? (2) Is there a preferred treatment pattern or combination of multiple medications? (3) Is there a treatment pattern that has a significantly better success rate compared to other treatment patterns? (4) What are the differences in efficacy between polypharmacy and monotherapy? (5) To what extent can polypharmacy be explained through the factors *clinical diagnosis*, *previous history*, *severity at baseline*, *age*, and *gender*? (6) Which adverse side effects or combinations of adverse side effects are more common than others, and which are rare? (7) Which adverse side effects can be linked to specific drugs or drug combinations? (8) To what extent can adverse side effects be predicted from clinical data such as *combination of concurrent drugs*, *diagnosis*, *previous history*, *acute symptomatology*, *severity at baseline*, *age*, and *gender*.

## Data material

This “naturalistic” longitudinal study was observational and comprised of 320 patients hospitalized at 3 residential mental health treatment centers with a clinical diagnosis of either schizophrenic (ICD-10: “F2x.x”; *n* = 94; “F2 patients”) or depressive disorders (ICD-10: “F3x.x”; *n* = 226; “F3 patients”). The patients were informed about the goals of this research project and that they can discontinue participation at any time without giving reasons and without facing any disadvantages from this.

The study protocol included (1) assessments of previous history and overall social functioning through the 63-item SADS Syndrome Check List SSCL-16 and 83-item SADS-Supplement SSCL-SUPP (lifetime versions) [12]; (2) up to 8 repeated measurements over 5 weeks assessing the time course of improvement through the 17/21-item Hamilton Depression Scale HAM-D [13] and the 30-item Positive and Negative Syndrome Scale PANSS [14]; (3) up to 8 repeated measurements over 5 weeks assessing medication and unwanted side effects through the 46-item Medication and Side Effects Inventory MEDIS [15]; and (4) the collection of blood samples for serum extraction and DNA isolation. The repeated assessments regarding the time course of improvement and unwanted side effects were carried out at weekly intervals plus 2 additional assessments at the 3rd and 10th study day.

The syndrome-oriented instrument SSCL-16 extends the ICD-10 definitions by replacing the yes–no dichotomy of diagnostic schemata by the dimensional quantities «schizophrenic thought disorders», «delusions», «hallucinations», «ego consciousness», «incongruent affect», «anergia», «depressive syndrome», «manic syndrome», and «suicide», while the SSCL-SUPP measures the patients’ overall level of functioning, social relations, affective lability, personality traits, somatization, and consumption behavior.

The HAM-D instrument assesses the severity of depressive disorders by means of a single scale, while the PANSS instrument assesses the severity of schizophrenic disorders in terms of positive, negative, and general psychopathology scales. The MEDIS instrument details side-effect clusters in a quantitative way with respect to «sleep», «appetite», «sexuality», «gastro-intestinal», «cardiac-respiratory», «autonomic», «psychosomatic», «neurological», and «cardiovascular» disturbances.^1^

**Fig. 1:**
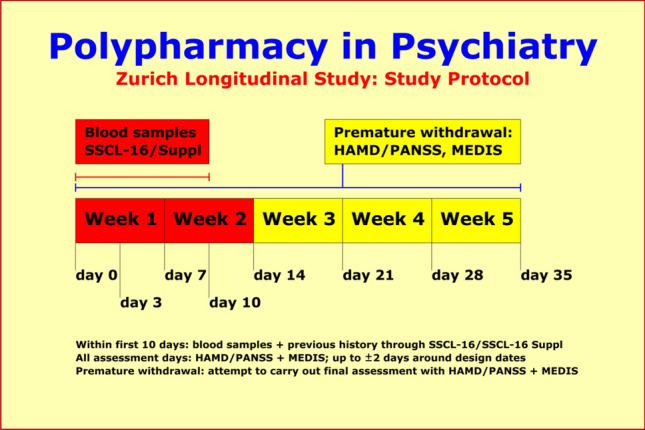
Our study protocol included (1) assessment of previous history and overall social functioning at entry into study; (2) repeated measurements of psychopathology, medication, and unwanted side effects over 5 weeks; and (3) the collection of blood samples

A minimum baseline score of at least 21 on the general psychopathology PANSS-G Scale^2^ (primary “F2x.x” diagnoses), or of at least 15 on the HAM-D17 Scale (primary “F3x.x” diagnoses), was required at entry into study. Patients were explicitly excluded if diagnosis was due to an organic background or psychoactive substance abuse. Based on these “naturalistic” criteria, about 70–80% of hospitalized patients were eligible for the study so that results are expected to be for the most part generalizable to psychiatric inpatients in other hospitals. We used all scales for all patients, even though no more than about 35% of F2 patients suffer from *significant* depressive symptoms, and no more than about 25% of F3 patients suffer from *significant* paranoid symptoms (Fig. 1).

## Methods

The patients’ characteristics were modeled using quantitative multi-dimensional profiles of psychopathology, previous history, the time course of improvement under therapy, along with the adverse side effects caused by therapeutic interventions. The respective data originated from observer ratings on the basis of the SSCL-16, SSCL-SUPP instruments (previous history), and the HAM-D, PANSS, and MEDIS instruments (response to therapeutic interventions, adverse side effects). The raw instrument data were summarized for each individual patient in terms of multidimensional syndrome and side effect scores. The side effect scores ***S***_***k***_ (*k* = *1,2,.. 9*) regarding «sleep», «appetite», «sexuality», «gastro-intestinal», «cardiac-respiratory», «autonomic», «psychosomatic», «neurological», and «cardiovascular» disturbances were stratified according to the following scheme: (1) no side effects: ***S***_***k***_ ≤ 10; (2) mild side effects: 10 < ***S***_***k***_ ≤ 30; (3) moderate side effects: 30 < ***S***_***k***_ ≤ 40; (4) severe side effects: 40 < ***S***_***k***_ ≤ 50; and (5) very severe side effects: 50 < ***S***_***k***_. Thus, each individual patient’s response to therapeutic interventions was assessed through a longitudinal profile encompassing up to 8 repeated HAM-D, PANSS, and MEDIS scores.

From our previous study of 2,848 patients comparing the onset of action of 7 different antidepressants (monotherapy) and placebo [2], we have learned that adverse side effects start with the beginning of the medication, reach their maximum on the 10th day of treatment, and then slowly subside, probably due to habituation effects. For this reason, we have chosen the 10th day of treatment as the reference date for the analysis of side effects.

**Fig. 2:**
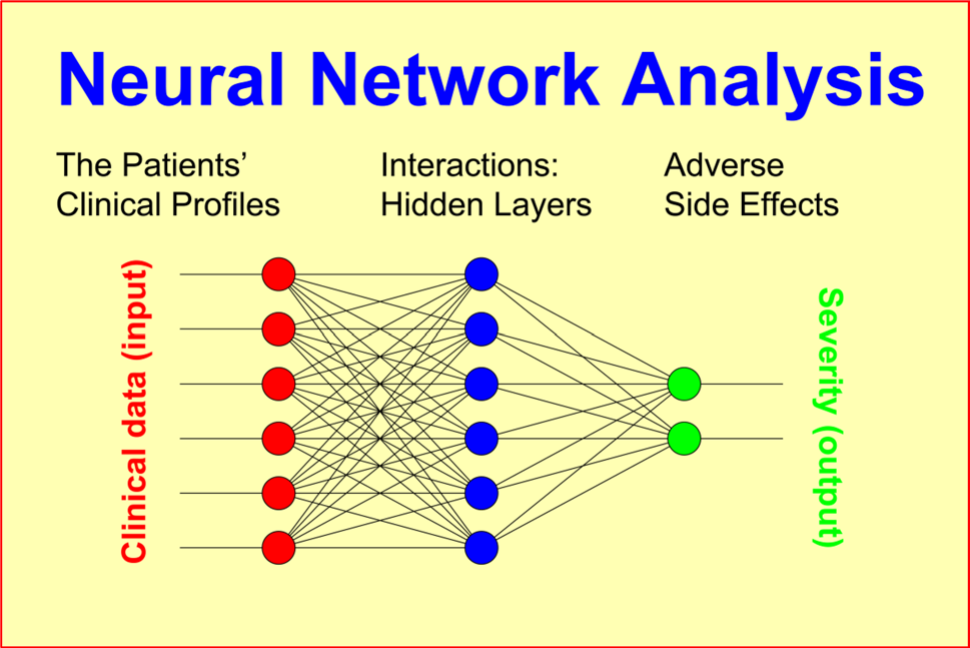
Principal schema of a neural net model where, for example, non-specific IgM levels result from multiple clinical and nonclinical factors connected to each other by complex interactions

In addition to the exploratory analyses which summarize the main data characteristics, we aimed to “learn” the development of adverse side effects as a function of the clinical data to set up prediction models by means of Neural Network analyses (NN) (Fig. 2).

**Table:** 

(i)	Output:	$s_{i} = \sigma \left[ {\sum\limits_{j}^{{}} {w_{ij} s_{j} } } \right]$	*s*_*i*_*: y*_*i*_ observed	(*i* = *1,2,… N*_i_)
(j)	Hidden layers:	$s_{j} = \sigma \left[ {\sum\limits_{k}^{{}} {w_{jk} s_{k} } } \right]$		(*j* = *1,2,… N*_j_)
(k)	Input:	$s_{k} = x_{k}$	*x*_*k*_ observed	(*k* = *1,2,… N*_k_)

In this methodological approach, “learning” means that for a suitable selection of clinical data such as *concurrent drugs*, *diagnosis*, *previous history*, *acute symptomatology*, *severity at baseline*, *age*, and *gender*, a NN model was iteratively determined that predicted the observed side effect scores in each patient as accurately as possible. Such a model must not necessarily exist since the hypothesized relationship between the model’s input and output may either not exist in principle, or may not exist uniformly for the sample as a whole entity.

Nonlinear NN models connect the “neurons” of input and output layers via one or more “hidden” layers, thus featuring a relatively large number of free parameters. NN connections are realized through (1) weight matrices and (2) model fitting algorithms minimizing an error function in the weight space (goodness of fit). All outputs are computed using sigmoid thresholding of the scalar product of the corresponding weight and input vectors. Outputs at stage “***s***” are connected to each input of stage “***s*** + 1”. The most popular model fitting strategy, the backpropagation algorithm, looks for the minimum of the error function using the method of gradient descent (“steepest descent”). The basic algorithm is:

**Table:** 

	Improvements:	$\Delta w_{ij} = \alpha \cdot \varepsilon_{i}^{\nu } \cdot s_{j} \cdot s_{i} (1 - s_{i} )$	$\varepsilon_{i}^{\nu } = y_{i}^{\nu } - s_{i}^{\nu }$	(*ν* = *1,2,.. p*)
		$$\Delta w_{jk} = \alpha \cdot \sum\limits_{i = 1}^{{N_{i} }} {\varepsilon_{i}^{\nu } } \cdot s_{k} \cdot s_{i} (1 - s_{i} ) \cdot w_{ij} \cdot s_{j} (1 - s_{j} )$$		

where ***x***_k_ denote observed stimuli, ***y***_j_ observed responses, σ the activation function of sigmoid-type: R → (0,1), ***α*** the learning rate, and ***p*** the number of probes (patients). The achievable precision of the model essentially depends on the information included, the quality of underlying data, and the number of intermediate layers implemented to model nonlinear interactions. The computational load, on the other hand, increases exponentially with the number of layers.

Results derived through standard NN approaches, which use 80% of samples for training and the remaining 20% for testing tend to be over-optimistic, in particular in the presence of assessment errors and missing data. By contrast, the *k*-fold cross-validation approach splits the data into ***k*** roughly equal parts, using ***k***-1 partitions for training, while one partition is used for testing. This process is repeated until each partition has served as a testing set, so that ***k*** estimates of prediction errors are generated. The resulting prediction errors are approximately unbiased for the “true” error for sufficiently large ***k*** (***k*** ≈ 10 is a typical value in practice). In consequence, we relied on the *k*-fold cross-validation strategy with ***k*** = 10 throughout the entire project and applied the well-proven "random walk" strategy to distinguish between local and global minima.

## Statistical analyses

We used the *Statistical Analysis Software SAS/STAT 9.3* by SAS Institute Inc. for repeated measurement analyses, and the *SPSS 25 Statistics Package* by IBM along with *PROC HPNEURAL* from *SAS Enterprise Miner 15.1* for Neural Net analyses.

## Ethics

The study was approved by the local ethics committees of the Canton of Zurich and the Canton of Thurgau. Written informed consent was obtained from all participants.

## Conflicts of interest

There are no conflicts of interest.

## Results

### Psychopathology

Of the 320 patients recruited within the scope of this longitudinal study, only 7 (2.2%) exercised their right to terminate their participation in the study at any time without giving any reason and without having any disadvantages. These patients dropped out in the first study week with 1–2 completed assessments, all with F3 diagnoses. They did not differ in any way from the other patients in the study.

There were 80 patients (25%) with rapid improvement (“rapid improvers”) so that they could be discharged from the hospital between the 15th and 20th day of therapy to continue treatment with their primary care physician (there was no assessment on the day of discharge). We found no differences between rapid improvers and the other patients in the study with two exceptions: (1) rapid F3 improvers had a significantly lower previous history burden (*p* = 0.0235); and (2) among the rapid F2 improvers, there were tendentiously more males than females (*p* = 0.1046). It was not possible to reliably predict rapid improvers by means of NN methods using sociodemographic data, baseline severity, and the 63 + 83 previous history items.

Totally 128 patients remained in the study until the envisaged end of the study period (“late improvers” or “non-improvers”): 43 F2 patients (45.7%) and 85 F3 patients (37.6%). They did not differ from the other patients and, again, it was not possible to find a reliable prediction model for them.

The sample included 142 males (mean age 39.6 ± 12.5 years) and 178 females (mean age 42.7 ± 12.5 years), distributed among the diagnostic groups as follows: F2 diagnoses with 47 males (mean age 37.5 ± 13.4 years) and 47 females (mean age 40.3 ± 11.2 years); F3 diagnoses with 95 males (mean age 40.7 ± 12.0 years) and 131 females (mean age 43.6 ± 12.8 years). The diagnostic groups did not differ in terms of education (p = 0.822) and age (*p* = 0.087).

As to the severity at baseline, the F2 patients exhibited a mean baseline score of 36.0 ± 8.70 on the PANSS-G scale: 20 mild cases (21.3%) with a PANSS-G baseline score < 30 (mean: 24.8 ± 3.11), 51 moderately ill cases (54.3%) with 30 ≤ PANSS-G baseline score ≤ 40 (mean: 34.5 ± 2.72), and 23 severely ill cases (24.4%) with a PANSS-G baseline score > 40 (mean: 47.6 ± 6.59). Regarding the HAM-D17 score, 31 patients (33.0%) reported moderate to severe depressive symptoms. By construction, these HAM-D17 items were closely related to the PANSS Depression Score (PDS), a fact which is reflected in a correlation coefficient of *r* = 0.557 (*p* < 0.0001). The mean PDS increased with the severity of the schizophrenic symptomatology as illustrated by a correlation coefficient of 0.549 (*p* < 0.0001): mild cases displayed a mean PDS of 8.2 ± 2.82, moderate cases a mean PDS of 10.5 ± 2.34, and severe cases a mean PDS of 14.2 ± 3.80. There were no gender differences in terms of illness severity, neither for previous history nor with respect to the acute symptomatology at baseline, with the only exception of a slightly higher PANSS depression score among women (*p* = 0.0463).

As to the F3 patients, we found for the total sample a mean baseline score of 23.0 ± 5.69 on the HAM-D17 Scale. The sample consisted of 62 mild cases (27.4%) with a HAM-D17 baseline score < 20 (mean: 15.9 ± 2.56), 79 moderately ill cases (35.0%) with a 20 ≤ HAM-D17 baseline score ≤ 24 (mean: 22.3 ± 1.27), and 85 severely ill cases (37.6%) with a HAM-D17 baseline score > 24 (mean: 28.8 ± 3.10). In terms of HAM-D21 items, 89 patients (39.4%) reported paranoid symptoms, predominantly delusions and hallucinations, and to a much lesser extent, depersonalization and de-realization. There were no gender differences in terms of illness severity, neither for previous history nor with respect to the acute symptomatology at baseline, with the only exception of a tendency towards a slightly higher score on HAM-D21 paranoid symptoms among women (*p* = 0.0647).

In summary, the group of F3 patients exhibited a similar symptomatic overlap across diagnoses as we had already seen for the F2 patients, thus underlining the so-called “continuum hypothesis” in psychiatry. In F3 patients, the severity of the depressive symptomatology significantly increased the severity of a “paranoid component” and, vice versa, in F2 patients, the severity of the schizophrenic symptomatology significantly increased the severity of the “depressive component”. In terms of psychopathological syndromes, clinical diagnoses did not represent entities that could be clearly distinguished from one another. The most distinctive syndrome between depressive and schizophrenic disorders was “Ego Consciousness”, involving symptoms such as depersonalization and de-realization: uncertainty of being oneself, feelings of strangeness or of having changed; delusional belief that one’s appearance, or an organ system, is diseased; feelings of being outside of one’s body; odd or bizarre ideation or magical thinking.

### Polypharmacy

Our data showed that polypharmacy was omnipresent. In clinical reality, polypharmacy apparently did not only mean the use of multiple medications concurrently, but it also seemed to involve poly-diversity regarding the selection of drugs. No less than 328 different medications (trade names) were used to treat the 320 study patients with a total of 236 different active compounds.

**Fig. 3:**
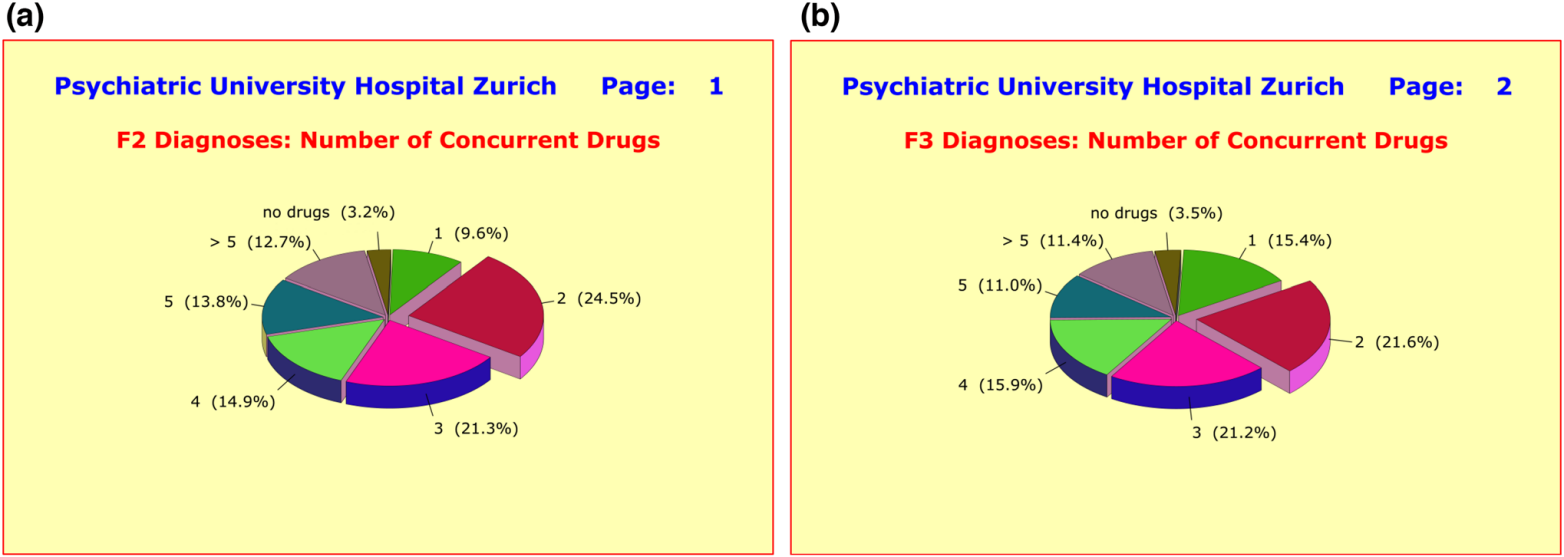
**a** Treatment regimen of 94 hospitalized patients treated for a clinical diagnosis of schizophrenic disorders (ICD10 F2). As little as 9.6% of patients were treated with monotherapy, while the vast majority (62.7%) received a combination of three or more concurrent drugs. The favored treatment regimen for patients with an F2 diagnosis appears to rely on two antipsychotics plus 1 antidepressant, supplemented by medications that compensate for adverse side effects. **b** Treatment regimen of 226 hospitalized patients treated for a clinical diagnosis of major depressive disorders (ICD10 F3). No more than 3.5% of patients were treated with psychotherapy alone, and 15.4% with monotherapy. The vast majority (59.5%) received a combination of three or more concurrent drugs. The favored treatment regimen for patients with an F3 diagnosis appeared to rely on 2 antidepressants plus 1 antipsychotic, supplemented by medications that compensate for adverse side effects

On average, the patients received 4.50 ± 2.68 medications,^3^ consisting of 3.30 ± 1.84 psychotropic drugs, plus 0.79 ± 1.13 medications that alleviate adverse side effects, plus 0.41 ± 0.89 other somatic medications. Psychotherapy without concurrent medication and monotherapy were with 2.7% and 9.7% (F2 patients) and 3.0% and 15.6% (F3 patients) rare exceptions. Only marginal differences showed up between F2 and F3 patients: 4.33 ± 1.98 versus 4.56 ± 2.69 concurrent medications (*p* = 0.3928) (Fig. 3a, b).

**Fig. 4:**
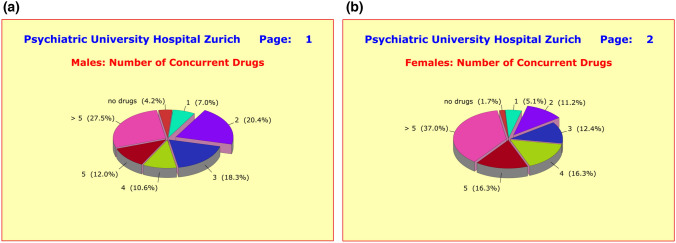
**a** Treatment regimen of 142 hospitalized male patients treated for a clinical diagnosis of either major depressive (ICD10 F3) or schizophrenic disorders (ICD10 F2). Male patients received with an average of 4.04 ± 2.43 concurrent drugs significantly fewer medications than female patients with 4.86 ± 2.50 concurrent drugs (*p* = 0.0032). **b** Treatment regimen of 178 hospitalized female patients treated for a clinical diagnosis of either major depressive (ICD10 F3) or schizophrenic disorders (ICD10 F2). Male patients received with an average of 4.04 ± 2.43 concurrent drugs significantly fewer medications than female patients with 4.86 ± 2.50 concurrent drugs (*p* = 0.0032)

By contrast, significant differences between the hospital wards in charge of treatment were detected. While two hospital wards followed virtually identical treatment regimens (4.82 ± 2.26 and 4.85 ± 3.32 concurrent medications), the wards of the third hospital showed significantly lower drug use (3.22 ± 2.01 concurrent medications; *p* = 0.0022). Female patients received with 4.86 ± 2.50 concurrent medications significantly more pharmacological treatment than male patients with 4.04 ± 2.43 concurrent medications (*p* = 0.0032). This difference could not be explained by the subtle differences in illness severity at baseline (Fig. 4a, b).

Given these figures, the question arises as to what factors guide clinicians in making decisions regarding the number of drugs used concurrently in the individual case. To address this question, we evaluated a linear prediction model that included the parameters «gender», «age», «diagnosis», «hospital», «previous history» in terms of lifetime occurrence of affective and schizophrenic symptoms, and «severity at baseline» in terms of PANSS-G and HAM-D17 baseline scores. This model did not explain more than 14.1% of the observed between-subject variance regarding medication regimen (number of concurrent medications). A tentative analysis of the parameter «psychiatrist in charge» resulted in a further 21.0% of explainable variance. Taken together, our analyses suggested that as much as 65% of the variance inherent in the polypharmacy treatment approach was just random.

**Fig. 5:**
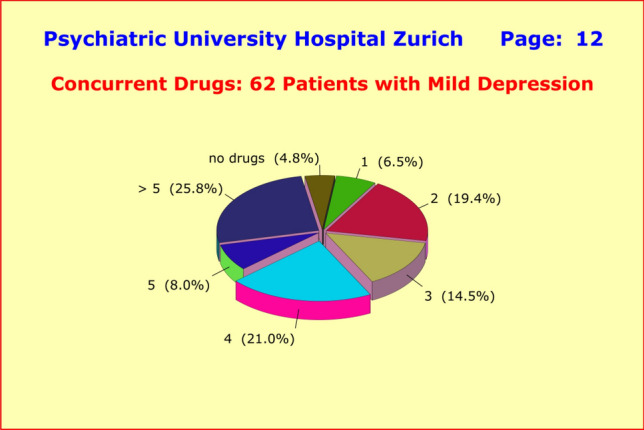
Treatment regimen of hospitalized 62 patients treated for a clinical diagnosis of major depressive disorders (ICD10 F3) and suffering from mild depression (HAM-D17 baseline score of 15–19). The figures demonstrate that polypharmacy is used ubiquitously, regardless of baseline severity at the start of treatment even though placebo-controlled drug trials rarely ever showed superiority of active compounds over placebo in mild depression

How little influence the parameter "severity at baseline" had, became evident in the observed treatment regimen of patients with mild depression (HAM-D17 baseline score of 15–19) for whom placebo-controlled drug trials rarely ever show superiority of active compounds over placebo [2]. Contrary to expectations, our analyses revealed virtually no differences in the medication regimens when compared with the treatment of moderately or severely ill cases (Fig. 5).

### Preferred drug combinations

We started the analysis of polypharmacy data with the expectation that there are a few preferred drug combinations that can easily be compared in terms of efficacy and side effects. But the sheer number of medications used by the treating psychiatrists of this study, —summing up to an average of 4.5 concurrent medications per patient, and amalgamated in numerous combinations—, has led to an enormous diversity of medication combinations. Even though some drug combinations were more common in the multitude of observed polypharmacy regimens, a clear and widely accepted strategy was not discernible (Table 1).

**Table 1:** Frequencies of adverse side effects in 94 hospitalized patients treated for a clinical diagnosis of schizophrenic disorders (ICD10 F2)

Percentage of F2 patients reporting adverse side effects			
	Day_01 (%)	Day_10 (%)	Severe forms (%)
Sexuality	41.77	41.35	32.91
Autonomic disturbances	43.98	43.15	21.83
Cardiac-respiratory disturbances	49.21	47.42	27.05
Psychosomatic disturbances	58.39	58.20	27.69
Appetite increased/decreased	60.60	59.28	41.30
Gastrointestinal disturbances	69.15	70.79	30.69
Neurological disturbances	72.94	72.13	22.00
Sleep disturbances	78.96	77.08	51.90
Cardiovascular disturbances	90.35	90.79	55.69

Adverse side effects were assessed by a specifically trained health professional on the basis of a catalog of 46 items covering 9 adverse side effect clusters (first column). The severity of an adverse reaction was rated as mild, moderate, severe, and very severe. The nine clusters are sorted in ascending order of frequency from 41.77% (sexuality) to 90.35% (cardiovascular disturbances). Percentages relate to the first 3 days of treatment (Day_01) and to the 10th day of treatment (Day_10), displaying only minor changes over time. The percentages of severe forms (rated as severe or very severe) are given in the last column

Rather, the common denominator underlying the treatment strategies appeared to be an almost “mandatory” combination of antidepressants with antipsychotics. We found that 42.6% of the F2 patients were treated in this way (9.6% received one antipsychotic plus at least one antidepressant; 33.0% received two or more antipsychotics plus at least one antidepressant). Similarly, with 49.1% approximately half of the F3 patients followed such a treatment regimen (19.9% received one antidepressant plus at least one antipsychotic; 29.2% received two or more antidepressants plus at least one antipsychotic). The addition of benzodiazepines as an adjunct treatment was another commonly used approach, intended to augment the efficacy of the primary medication. This option was chosen in almost one third of the cases, very similarly for both F2 (28.7%) and F3 (29.2%) patients.

### Response to treatment

In line with our previous studies in this field (cf. [2, 17]), we used scale-based cutoff values for the definition of response to treatment. Response under depression therapies was defined by a 50% HAM-D17 baseline score reduction and response under schizophrenia therapies by a 40% PANSS-P baseline score reduction.^4^ Similarly, we defined “improvement” by a sustained 20% HAM-D17, or a 20% PANSS-P baseline score reduction, respectively, so that we were able to analyze the relationship between early improvement and later response [18, 19].

Response to acute treatment under a polypharmacy regimen was generally modest with response rates of 25.5% for the F2 patients, and 36.7% for the F3 patients (mild cases: 33.9%; moderately ill cases: 35.0%; severely ill cases: 37.6%). The response rates did not differ between the hospitals in charge (*p* = 0.9145). Most improvement occurred within the first 2 weeks of treatment. Of the F2 patients, 44.7% showed improvement within the first 12 days, and 48.9% within the first 2 weeks. Of the F3 patients, 65.9% showed improvement within the first 12 days and 70.0% within the first 2 weeks. For the F3 patients under monotherapy or psychotherapy alone, the responder rate was 44.2%. The observed outcome differences between polypharmacy and monotherapy did not reach statistical significance (*p* = 0.1559). The results of the data analyses, however, left no doubt whatsoever that (1) compared to monotherapy, the various polypharmacy regimens did not provide any advantage to patients in terms of efficacy; and (2) with 36.7% for F3 patients and 25.5% for F2 patients, the responder rates exhibited a general drop of about 40% compared to what was the standard 20 years ago. For example, in a comparison between *olanzapine* and *haloperidol* [6], or in a comparison of the five antidepressants *Imipramine* (60.9%), *Moclobemide* (53.1%), *Fluoxetine* (47.5%), *Mirtazapine* (56.5%), and *Paroxetine* (53.0%) [5].

### Adverse side effects

**Fig. 6:**
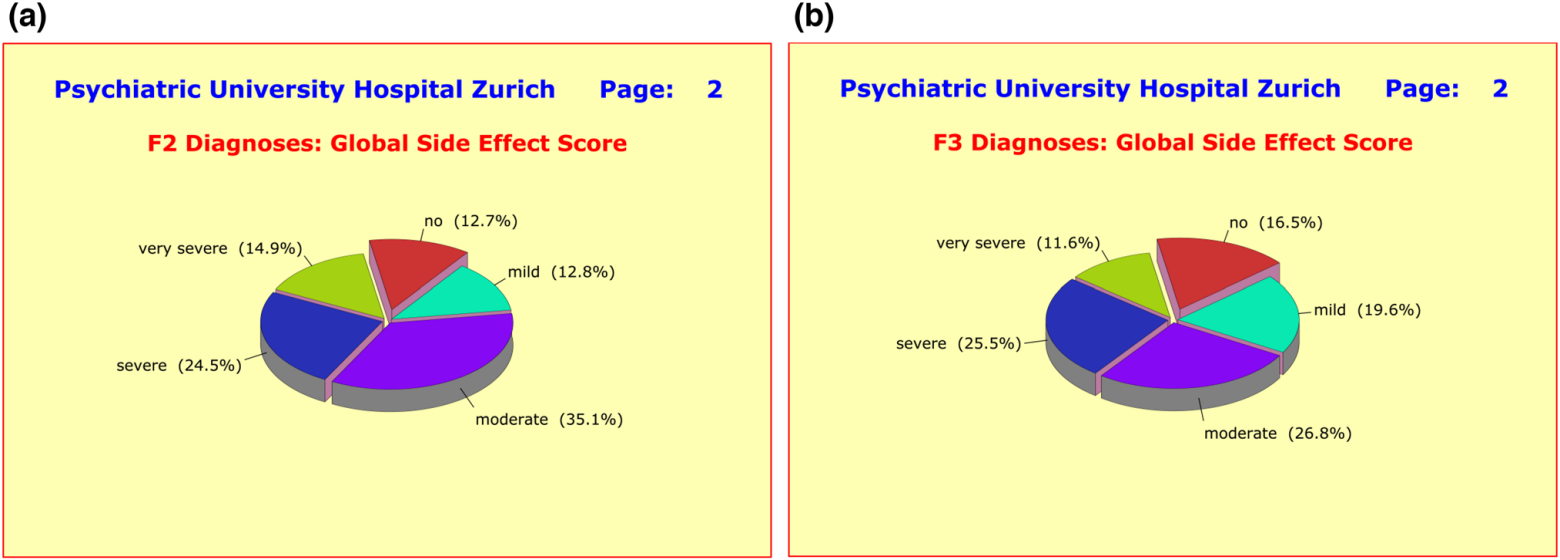
**a** As precursor to the prospective therapeutic benefits and as permanent companion to therapy, most patients experienced significant adverse side effects induced by their psychotropic medications. In terms of the global side effect score, as many as 87.3% of the F2 patients reported at least “some” mild adverse effects, of which 39.4% were of severe or very severe form. The differences to F3 patients were marginal. **b** As precursor to the prospective therapeutic benefits and as permanent companion to therapy, most patients experienced significant adverse side effects induced by their psychotropic medications. In terms of the global side effect score, as many as 83.5% of the F3 patients reported at least “some” mild adverse effects, of which 37.1% were of severe or very severe form. The differences to F2 patients were marginal

As precursor to the prospective therapeutic benefits and as a permanent companion to psychopharmacological treatments, most patients experienced significant adverse side effects induced by their psychotropic medications. In terms of global side effect scores, as many as 87.3% of the F2 patients (Fig. 6a) and 83.5% of the F3 patients (Fig. 6b) reported at least “some” mild adverse effects. The percentages of the severe forms were 39.4% (F2) and 37.1% (F3), and for the mild to moderate forms 47.9% (F2) and 46.4% (F3). The differences between the diagnostic groups were generally small and did not reach statistical significance (*p* = 0.3373). However, females reported more severe side effects than males (42.1 vs 32.1%; *p* = 0.0258), which is most likely due to the fact that female patients received with 4.86 ± 2.50 concurrent medications significantly more pharmacological treatment than male patients (4.04 ± 2.43 concurrent medications). Expectedly, the global side effect score was found to be closely linked with the number of concurrent medications (*p* = 0.0005). It was therefore not surprising that patients under monotherapy experienced only a fraction of the side effects compared to patients under polypharmacy (*p* = 0.0112). For example, in the case of neurological disturbances, just 1 patient (5.3%) under monotherapy compared to 103 patients under polypharmacy (35.3%), in the case of cardiovascular disturbances just 2 patients (10.5%) under monotherapy compared to 97 patients under polypharmacy (33.2%), and regarding the global side effect score just 2 patients (10.5%) under monotherapy compared to 117 patients under polypharmacy (40.1%).

By construction, global side effect scores are too unspecific and insufficiently sensitive to measure the differences between the diagnostic groups with regard to adverse side effects, or to assess the contributions of single drugs to the development of specific adverse side effects. Rather, we relied on the 46 side effect items of the MEDIS instrument, which cover the following 9 side effect clusters: «sleep», «appetite», and «sexuality», along with «gastro-intestinal», «cardiac-respiratory», «autonomic», «psychosomatic», «neurological», and «cardiovascular» disturbances. The MEDIS items were assessed by a specifically trained psychiatrist and psychologists to improve inter-rater agreement.

This splitting of the multiplex side effect profiles into several intrinsic sub-clusters not only affirmed considerable differences between the sub-clusters in terms of their occurrence, but also revealed some highly significant differences between the diagnostic groups although the majority of side-effect clusters were very similar across diagnoses. Among the F2 patients, the observed incidences reached from 41.8% (sexuality) to 90.4% (cardiovascular disturbances), and among the F3 patients from 41.3% (neurological disturbances) to 79.4% (sleep). All side-effect clusters were quite stable over time and seemed to be virtually unchanged on day 10 of the study when compared with the scores of the first assessment (Tables 1, 2).

**Table 2:** Frequencies of adverse side effects in 226 hospitalized patients treated for a clinical diagnosis of major depressive disorders (ICD10 F3)

Percentage of F3 patients reporting adverse side effects			
	Day_01 (%)	Day_10 (%)	Severe forms (%)
Neurological disturbances	41.31	39.96	9.31
Sexuality	49.10	50.78	41.45
Cardiac-respiratory disturbances	50.41	49.03	25.44
Autonomic disturbances	54.90	55.17	26.00
Appetite increased/decreased	56.34	56.73	35.10
Psychosomatic disturbances	61.52	60.33	30.90
Gastrointestinal disturbances	67.17	66.37	26.34
Cardiovascular disturbances	71.31	72.32	36.41
Sleep	79.38	78.95	56.83

Adverse side effects were assessed by a specifically trained health professional on the basis of a catalog of 46 items covering 9 adverse side effect clusters (first column). The severity of an adverse reaction was rated as *mild*, *moderate*, *severe*, and *very severe*. The nine clusters are sorted in ascending order of frequency from 41.31% (neurological disturbances) to 79.38% (sleep). Percentages relate to the first 3 days of treatment (Day_01) and to the 10th day of treatment (Day_10), displaying only minor changes over time. The percentages of severe forms (rated as severe or very severe) are given in the last column

**Fig. 7:**
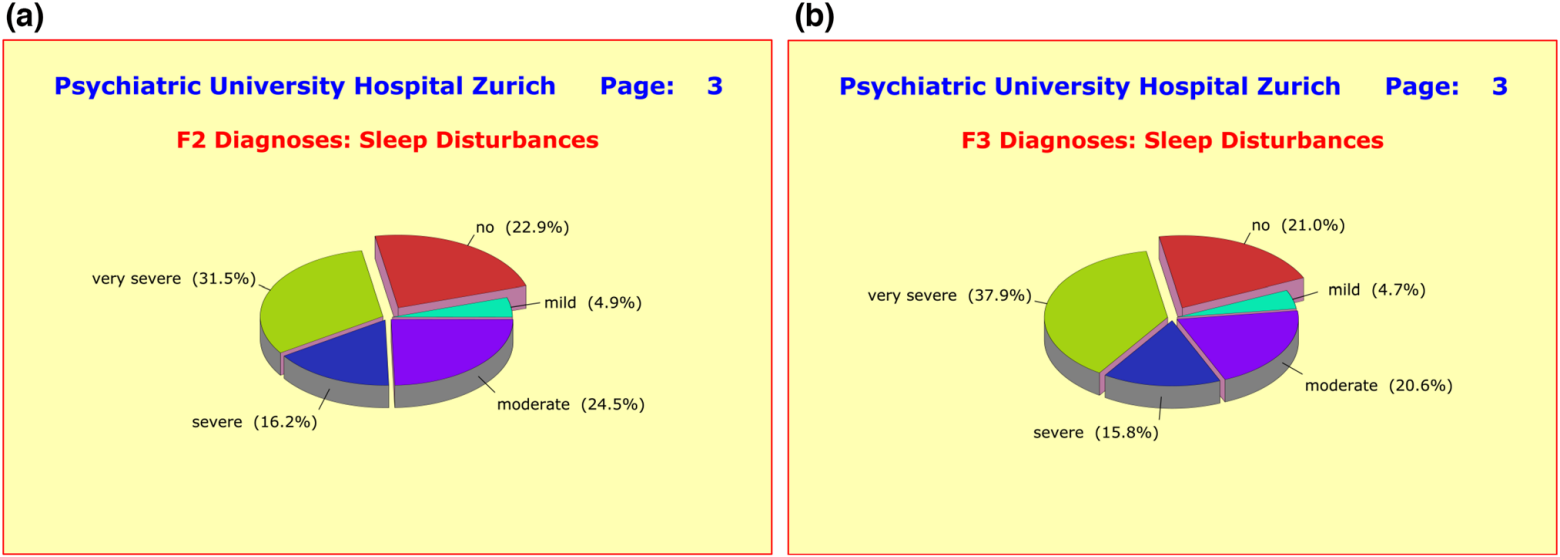
**a** Grouped into nine clusters (*sleep*, *appetite*, *sexuality*, and *gastro-intestinal*, *cardiac-respiratory*, *autonomic*, *psychosomatic*, *neurological*, and *cardiovascular disturbances*) the majority of adverse effects showed little to no difference between diagnostic groups, presumably due to the fact that patients received an average of 4.50 ± 2.68 medications irrespective of primary diagnosis. With regard to sleep disturbances, the differences to F3 patients were small and clinically irrelevant. **b** Grouped into nine clusters (*sleep*, *appetite*, *sexuality*, and *gastro-intestinal*, *cardiac-respiratory*, *autonomic*, *psychosomatic*, *neurological*, and *cardiovascular disturbances*) the majority of adverse effects showed little to no difference between diagnostic groups, presumably due to the fact that patients received an average of 4.50 ± 2.68 medications irrespective of primary diagnosis. With regard to sleep disturbances, the differences to F2 patients were small and clinically irrelevant

**Fig. 8:**
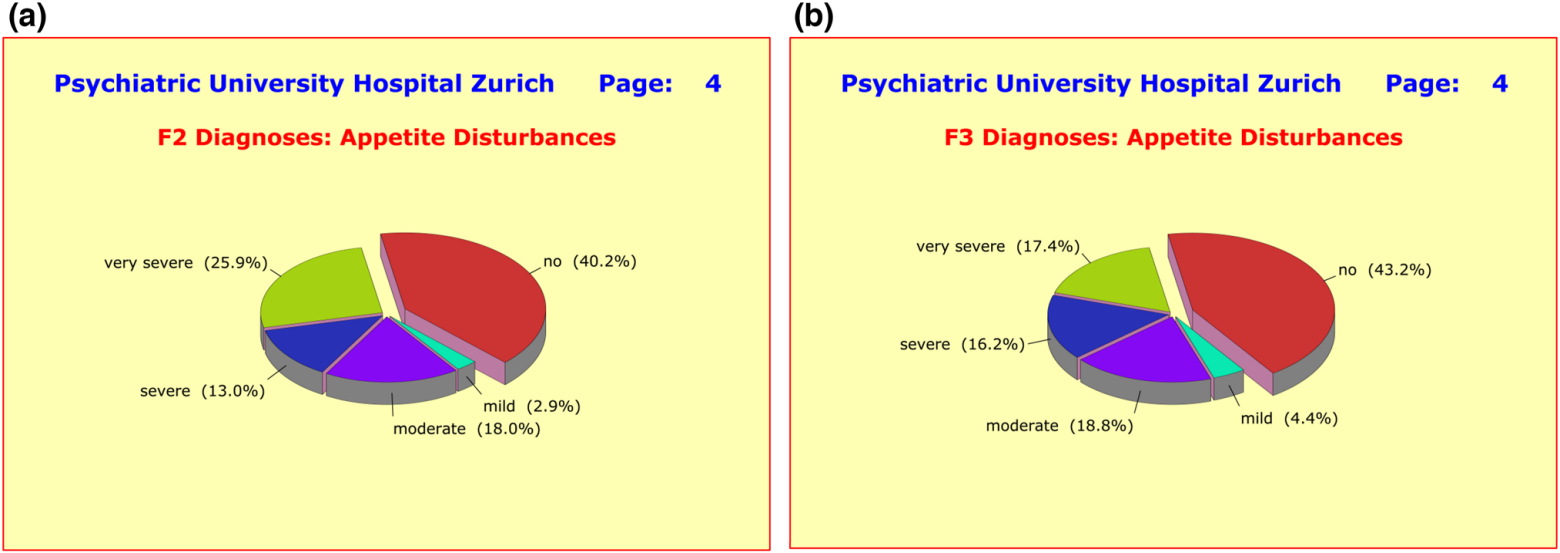
**a** Grouped into nine clusters (*sleep*, *appetite*, *sexuality*, and *gastro-intestinal*, *cardiac-respiratory*, *autonomic*, *psychosomatic*, *neurological*, and *cardiovascular disturbances*) the majority of adverse effects showed little to no difference between diagnostic groups, presumably due to the fact that patients received an average of 4.50 ± 2.68 medications irrespective of primary diagnosis. With regard to appetite disturbances (weight gain), the differences to F3 patients were generally small yet highly significant for the very severe forms (F2: 25.9% vs. F3: 17.4%). **b** Grouped into nine clusters (*sleep*, *appetite*, *sexuality*, and *gastro-intestinal*, *cardiac-respiratory*, *autonomic*, *psychosomatic*, *neurological*, and *cardiovascular disturbances*) the majority of adverse effects showed little to no difference between diagnostic groups, presumably due to the fact that patients received an average of 4.50 ± 2.68 medications irrespective of primary diagnosis. With regard to appetite disturbances (weight gain), the differences to F2 patients were generally small yet highly significant for the very severe forms (F2: 25.9 vs. F3: 17.4%)

**Fig. 9:**
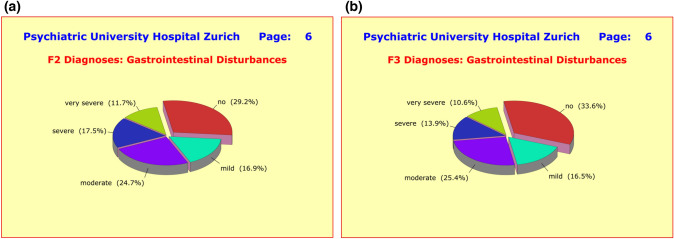
**a** Grouped into nine clusters (*sleep*, *appetite*, *sexuality*, and *gastro-intestinal*, *cardiac-respiratory*, *autonomic*, *psychosomatic*, *neurological*, and *cardiovascular disturbances*) the majority of adverse effects showed little to no difference between diagnostic groups, presumably due to the fact that patients received an average of 4.50 ± 2.68 medications irrespective of primary diagnosis. With regard to gastrointestinal disturbances, the differences to F3 patients were small and clinically irrelevant. **b** Grouped into nine clusters (*sleep*, *appetite*, *sexuality*, and *gastro-intestinal*, *cardiac-respiratory*, *autonomic*, *psychosomatic*, *neurological*, and *cardiovascular disturbances*) the majority of adverse effects showed little to no difference between diagnostic groups, presumably due to the fact that patients received an average of 4.50 ± 2.68 medications irrespective of primary diagnosis. With regard to gastrointestinal disturbances, the differences to F2 patients were small and clinically irrelevant

As mentioned before, the majority of side-effect clusters were very similar in both diagnostic groups. For example, 79.0% of the F2 patients and 79.4% of the F3 patients complained of sleep disturbances such as *difficulties falling asleep*, *interrupted or shortened sleep*, *early wakening*, and *drowsiness throughout day*. Of these patients, 51.9%/56.8% reported severe forms of sleep disturbances (Fig. 7a, b). Regarding appetite and weight gain, similar numbers of patients in both diagnostic groups were affected (Fig. 8a, b). Also noteworthy is the similarity of the incidences of gastrointestinal disturbances, such as *nausea*, *vomiting*, *gastric discomfort*, *constipation*, *diarrhea*, *hypersalivation*, or *dry mouth* (Fig. 9a, b).

**Fig. 10:**
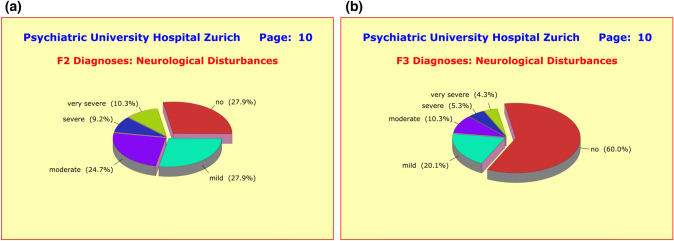
**a** In contrast to the majority of the nine side effect clusters under investigation, where only small and clinically irrelevant differences showed up between the diagnostic groups, marked differences were found between F2 and F3 patients in terms of neurological disturbances. In the F2 group, no less than 72.1% of patients reported at least “mild” adverse effects compared to only 40% among the F3 patients. This difference is most likely due to the fact that most F2 patients receive two or more antipsychotics (along with one antidepressant), while F3 patients receive two antidepressants and only one antipsychotic. **b** In contrast to the majority of the nine side effect clusters under investigation, where only small and clinically irrelevant differences showed up between the diagnostic groups, marked differences were found between F2 and F3 patients in terms of neurological disturbances. In the F2 group, no less than 72.1% of patients reported at least “mild” adverse effects compared to only 40% among the F3 patients. This difference is most likely due to the fact that most F2 patients receive two or more antipsychotics (along with one antidepressant), while F3 patients receive two antidepressants and only one antipsychotic

**Fig. 11:**
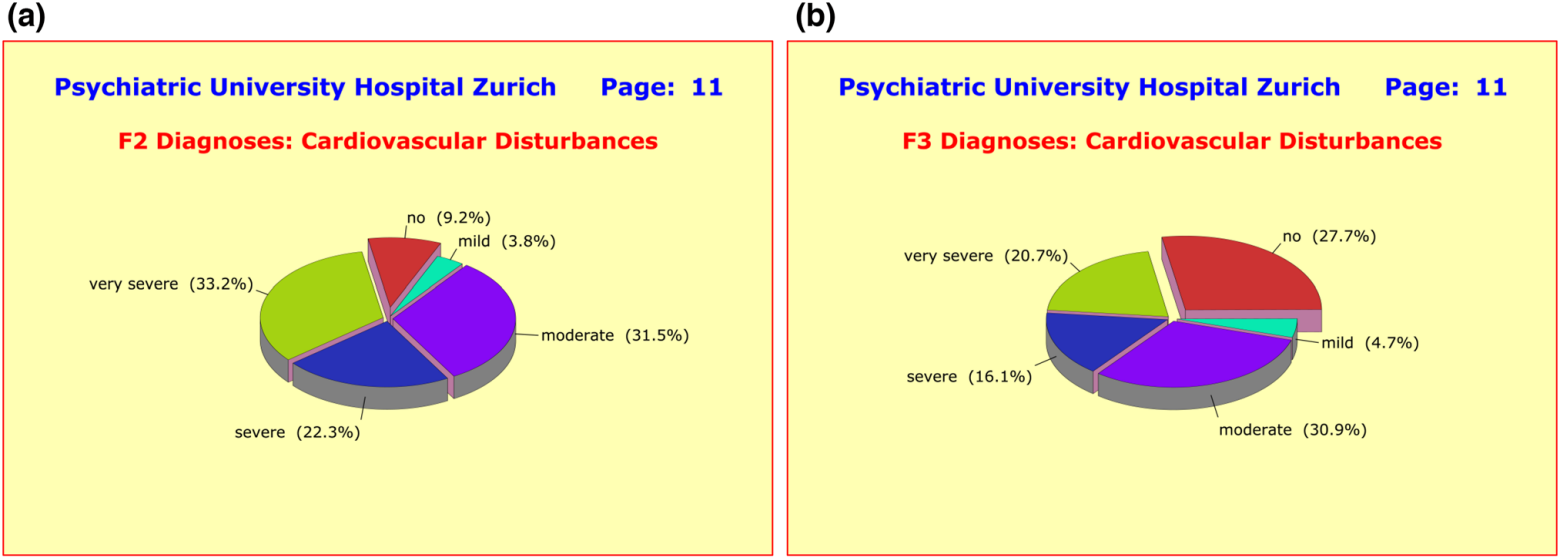
**a** In contrast to the majority of the nine side effect clusters under investigation, where only small and clinically irrelevant differences showed up between the diagnostic groups, marked differences were found between F2 and F3 patients in terms of cardiovascular disturbances. In the F2 group, no less than 87.0% of patients reported at least “mild” adverse effects compared to only 72.3% among the F3 patients. This difference is most likely due to the fact that most F2 patients receive 2 or more antipsychotics (along with one antidepressant), while F3 patients receive two antidepressants and only one antipsychotic. **b** In contrast to the majority of the nine side effect clusters under investigation, where only small and clinically irrelevant differences showed up between the diagnostic groups, marked differences were found between F2 and F3 patients in terms of cardiovascular disturbances. In the F2 group, no less than 87.0% of patients reported at least “mild” adverse effects compared to only 72.3% among the F3 patients. This difference is most likely due to the fact that most F2 patients receive two or more antipsychotics (along with one antidepressant), while F3 patients receive two antidepressants and only one antipsychotic

As one would have expected, there were huge differences between the diagnostic groups in terms of neurological and cardiovascular side effects, presumably due to the fact that F2 patients often take a combination of two (or more) antipsychotics which is rather rare among F3 patients. These adverse side effects included the neurological disturbances *hypertonia*, *hypotonia*, *tremor*, *acute dyskinesia*, *hypokinesis*, *akathisia*, *ataxia*, *nystagmus*, and *paresthesia* (Fig. 10a, b); as well as the cardiovascular disturbances *orthostatic hypotension*, *hypertension*, dysrhythmia, and *changes of blood count* (Fig. 11a, b).

Given the high, widespread incidence of serious adverse reactions, we aimed to identify those drugs (or combinations of drugs) that contributed most to the development and progression of severe side effect patterns. However, due to the large number of drugs used and the even larger number of drug combinations, this attempt proved to be extremely complex and not realizable in practice. In a somewhat stripped-down approach, we therefore focused our interest on patients with severe or very severe adverse side effects in the clusters «neurological disturbances» (NEURO: *n* = 49; 19 males and 30 females; 26 F2 patients and 23 F3 patients) and «cardiovascular disturbances» (CARDIO: *n* = 138; 58 males and 80 females; 51 F2 patients and 87 F3 patients).

The patients of the NEURO cluster received a total of 88 different medications with 75 different active substances (52 different psychoactive drugs with 42 different active substances resulting in a mean value of 3.84 ± 1.59 concurrent psychoactive drugs). Each of these drugs was administered in less than 10% of patients, and the number of two-drug combinations turned out to be as high as 327, each with an empirical frequency of < 2%. The situation for the CARDIO cluster was similar. The patients received a total of 149 different medications with 122 different active substances (72 different psychoactive drugs with 55 different active substances accumulating in a mean value of 3.54 ± 1.68 concurrent psychoactive drugs). Again, each of these drugs was administered in less than 10% of patients, and the number of two-drug combinations was with 811 again very high, each with an empirical frequency of < 1.5%.

To reduce this unmanageable treatment heterogeneity, we limited ourselves to the 12 most commonly used drugs and included them in a linear model in combination with *age*, *gender*, *diagnostic group*, *previous history*, *severity at baseline*, and *number of concurrent drugs*. In this way, a model could be found that explained 40% of the observed side effects of the NEURO cluster. The factors *age*, *gender*, *diagnostic group*, *previous history*, and *number of concurrent drugs* contributed most to the explainable variance, whereas the 12 selected drugs did not add more than 4% in the total. Of these 12 drugs, only the contribution of the active compound *Quetiapine* reached statistical significance, thus suggesting that it may have contributed significantly to the development of the side-effect clusters under investigation. On the other hand, the distinct heterogeneity of treatment modalities made it also clear that the adverse side effects observed in this study were most likely attributable to an unspecific overall drug “load” created in a variety of ways by the different drug combinations in use. This of course would presuppose a corresponding biological vulnerability on the part of patients.

No such model could be found for the side effect patterns of the CARDIO cluster, where the proportion of explainable variance did not exceed 10%. The insufficient model fit could again be due to an unspecific drug load as main causative factor, which is insufficiently covered by the mere number of concurrent drugs only. Subsequent analyses for all other side effect clusters were similarly unsuccessful in fitting linear models that explained a larger proportion of the observed variance. One exception was the cluster «psychosomatic disturbances» but also only with a modest 15.6% amount of explainable variance and no statistically significant contribution of pharmacological substances.

The three patients who had developed severe side effects under monotherapy were treated with the psychoactive substances *Quetiapine*, *Escitalopram*, or *Mirtazapine*.

### Predicting side effect patterns by Neural Net analysis

Given the modest performance of linear models in predicting and explaining the adverse side effects in this patient sample, our hopes now lay entirely in the non-linear models of Neural Net methods. A central problem, however, was again the large number of medications administered in this study, all potentially linked to the observed adverse reactions. To overcome this difficulty, we limited analyses to the 40 most common drugs of this study (21 antipsychotics, 19 antidepressants) and constructed 40-dimensional "medication vectors" into which dosages were entered for each individual patient at the appropriate positions and "0" elsewhere. Thus, the so-constructed medication vectors reflected the patients’ medication profiles in terms of the 40 most common drugs.

The aim was to predict the side effect patterns of each individual patient for day 10 of the therapeutic intervention, taking into account *age*, *gender*, *diagnostic group*, *previous history*, *severity at baseline*, *number of concurrent drugs*, and the *40-dimensional medication vector* as independent variables (“input”). It soon turned out that the initial goal of evaluating all nine side effect clusters simultaneously was not realizable on the basis of the available data. The prediction model was therefore reduced to the three clusters of *neurological*, *psychosomatic*, and c*ardiovascular* adverse side effects as dependent variables (“output”).

Despite the evaluation of a large number of competitive models, none could be found that fitted the empirical data sufficiently well. In particular, the percentage of correctly predicted side effects could not be brought much above 60%. However, common to all models was that the largest relative weight arose for the variables “number of concurrent drugs” (41.8%), “diagnostic group” (24.1%), and “gender” (18.2%). By contrast, the relative weight of the medication profile was consistently very small in the range of 1–3% while poorly reproducible. The relative importance of the variable “diagnostic group” had no "natural" biological basis, but was mainly due to the fact that psychiatrists tended to treat F2 patients with 2 antipsychotics plus 1 antidepressant, whereas F3 patients tended to receive just 1 antipsychotic plus 2 antidepressants. Similarly, the relative importance of the variable “gender” also appeared to be of a purely technical nature, due to the fact that women generally received more medication than men at comparable illness severities.

The results of the non-linear neural net analyses made it clear that adverse side effects can hardly be attributed to a specific drug or combination of drugs because patients are treated far too diversely. Rather, it seemed to be a certain level of unspecific “drug load”, along with a corresponding biological vulnerability of patients, that led to severe adverse reactions, largely independent of the actual pharmacological composition of the treatment regimen in the individual patient. Consequently, it is pretty unlikely that there can be a tailor-made polypharmacy regimen with minimum side effects for each individual patient.

On the other hand, it was interesting to see that a specifically trained neural net was able to predict the number of 0–5 concurrent psychotropic drugs quite reliably for each individual patient from his/her nine side effect clusters at a rate of 85%-90% correctly classified patients. Because of the distinct heterogeneity of medication regimens in psychiatry, however, the classifiers derived from our patient sample may not work equally well for other patient samples.

This latter result, which reverses the questions addressed by our study, emphasized again the dominant role of concurrency of multiple medications in the development of adverse side effects, but this kind of analyses is of course nothing more than an academic exercise with no further clinical relevance.

### Analysis of blood samples

The serum was used for the quantification and analysis of the unspecific, “natural” antibody immunoglobulin M (IgM). This followed our study on the role of inflammatory effects in the pathogenesis of psychiatric disorders based on monozygotic twins concordant and discordant for schizophrenic disorders [20]. The results of this analysis have already been published separately [6].

As part of a larger sample (*n* = 756) the patients' DNAs were genotyped for 91 specifically selected candidate genes, each with 5–8 polymorphic SNPs (totally 470 SNPs), along with 183 additional polymorphisms. The SNPs within each gene were combined into multi-dimensional, highly informative “vectors” and evaluated by means of NN methods and machine learning techniques.

The models used in this context rely on the results of our study with 2848 patients under 7 different antidepressants (monotherapy) and placebo [2]. These results had suggested that active substances, irrespective of their primary sites of action, act completely nonspecifically and only ***trigger*** recovery when they bring a complex, self-regulating system back into self-regulation and support it for a certain time. For antidepressants, it was possible in this way to separate early improvers (within the first 2 weeks) from late improvers (after 2 weeks). For antipsychotics, the results point in the same direction. All related analyses are still ongoing.

## Discussion

In this observational study, we aimed to assess today’s acute inpatient treatment of major depressive and schizophrenic disorders regarding therapeutic strategies, medications, adverse side effects, time course of recovery, and efficacy of treatments. Of particular interest was the weighing of the benefits and drawbacks of the polypharmacy approach. Results showed that polypharmacy is the treatment regimen of choice for psychiatric patients, while monotherapy and psychotherapy without supplemental psychotropic medication no longer appear to be treatment options.

The use of multiple medications can in certain cases be the appropriate and necessary therapeutic option [21–24]. For F3 patients, however, there are no reliable data in the literature comparing monotherapy with polypharmacy in terms of efficacy and adverse side effects [25, 26]. As to the treatment of F2 patients, several authors pointed out that “antipsychotic polypharmacy can work for some clinically difficult conditions but should be the exception rather than the rule and may be avoidable in many patients” [27], this in accordance with the guidelines [28]. In the respective publications “antipsychotic polypharmacy” is understood as a combination of just two antipsychotics with different pharmacodynamics actions. By contrast, in today's clinical reality “polypharmacy” means the combination of several antidepressants, antipsychotics, mood stabilizers, anxiolytics, hypnotics, antihistamines, and anticholinergics, along with other somatic treatments. As a direct consequence, the average patient of this study received 4.50 ± 2.68 medications, consisting of 3.30 ± 1.84 psychotropic drugs, plus 0.79 ± 1.13 medications that alleviate adverse side effects, plus 0.41 ± 0.89 other somatic medications. In particular, one rarely ever finds patients receiving less than two or more medications—even among cases with mild depression.

Contributing to a good deal to this development is the widespread pre-treatment of patients with antidepressants and antipsychotics by the family doctors so that drug-naïve patients are hardly ever seen anymore, neither among hospitalized nor outpatients. In other words, polypharmacy is ubiquitous in the acute treatment of depressive and schizophrenic disorders. Furthermore, we have to deal with the apparent contradiction between “mild depression” and “hospitalization”. Only a few patients with mild depression would actually need to be hospitalized and could certainly be treated successfully in other ways. However, the trend in psychiatry over the past two decades has been that (1) just every patient is treated straightaway with a combination of several antidepressants and antipsychotics; and (2) hospitalizations have become more frequent than strictly necessary.

One might argue that the polypharmacy approach is to a certain extent supported by the "recommendations" of the corresponding guidelines. However, our study suggests that the recommendations are interpreted in a way that is far too biased towards polypharmacy: a total of 118 F3 patients (52.2%) received 1–3 antipsychotics in addition to one or more antidepressants. Of these patients, 88 (74.6%) had no psychotic symptoms whereas 14 patients (6.2%) received no antipsychotics despite psychotic symptoms. Similarly, among the F2 patients, 75.5% received two or more antipsychotics and 46.8% received one or more antidepressants. The bottom line is that the observed polypharmacy practice can be explained only to a minor extent through the recommendations of the guidelines.

Given the very complexities of current mental health care, it is not really surprising that our study could not identify a rational strategy in the diversity of polypharmacy regimens that would have been widely accepted by psychiatrists. Rather, we learned from this study that “polypharmacy” is apparently also seen by psychiatrists as a way to clearly differentiate themselves from fellow psychiatrists. The 328 medications used to treat the 320 study patients in a sheer multitude of different combinations, and involving as many as 236 different active compounds, clearly point in this direction.

Most surprisingly, the “psychiatrist in charge of treatment” was found to be the main determining factor for a chosen treatment regimen, followed by gender, age, and diagnosis with much lesser weights. The severity of acute symptomatology at baseline played a negligible role in this context. The common denominator underlying the majority of treatment approaches of this study appeared to be that F2 patients have to be treated with 2 antipsychotics plus 1 antidepressant, and F3 patients with 2 antidepressants plus 1 antipsychotic.

By design, the results of this study relate to the acute treatment of patients with depressive or schizophrenic disorders. The situation may be somewhat different for the maintenance treatment of patients with schizophrenic disorders where the combination of two antipsychotics can in fact have beneficial effects as reported by a recent nationwide Finish cohort study of 62,250 individuals with schizophrenia and up to 20-year follow-up [23]. But overall, the evidence of a more favorable long-term outcome in maintenance treatments with a combination of two antipsychotics is scarce as suggested by a comprehensive Cochrane review study [11].

Adverse reactions are an inherent component of polypharmacy and tend to be much more severe than with monotherapy. Direct comparison between polypharmacy and monotherapy regimens revealed a 2–3 times higher incidence of severe adverse reactions under polypharmacy. Given the immense variety of different drug combinations observed in this study (rarely was the same treatment regimen used in more than two or three patients) adverse reactions could not be attributed with sufficient certainty to a particular drug or drug combination. On the contrary, the data gave us the impression that such adverse reactions could be triggered by virtually all drug combinations, depending on the patients’ vulnerability in this respect. Such a hypothesis would particularly explain the similarities in the distribution of side effect patterns across the diagnostic groups. The only exception was the side effect cluster of neurological disturbances, where a link with certain combinations of antipsychotics was detected.

Adverse side effects are experienced very subjectively by patients, depending on age, gender, general state of health, ability to suffer, as well as genetic makeup. There is no objective laboratory method that allows one to assess the kind and severity of adverse side effects regarding «sleep», «appetite», and «sexuality», or in terms of «gastro-intestinal», «cardiac-respiratory», «autonomic», and «psychosomatic» disturbances. Therapeutic drug monitoring methods are not really suitable for this purpose, especially when polypharmacy is involved and drug-drug interactions have a major impact on the side effect profile. Not to mention ethical concerns, since the cost–benefit ratio hardly justifies regular blood collection at pre-specified times on each rating day.

We therefore took a different approach to ensure, at least to some extent, that the adverse side effects reported by patients were due to the drugs involved: if adverse side effects are in fact closely related to the medications taken, then one can expect (1) a close correlation between the number of concurrent medications taken and the global adverse side effect score; and (2) significant differences in the side effect profiles between F2 and F3 patients. Both were shown to be the case in this study, so we can assume that a major component of the reported side effects was indeed due to the medications taken.

The significant increase in severe side effects, as well as the fact that polypharmacy does not result in a better therapy response, clearly speak against the widespread use of the polypharmacy approach in the treatment of hospitalized patients with a depressive or schizophrenic disorders. An exception may be patients for whom clozapine treatment would be advised, and for whom a combination of two adequate antipsychotics can have similar efficacy with significantly fewer severe side effects.

All in all, the results of this study have made it clear that the polypharmacy approach to treating depressive or schizophrenic patients can in no way, not even rudimentarily solve the problem that there is no causal therapy in psychiatry. Antidepressants and antipsychotics that differ greatly in their biochemical design and primary site of pharmacological action display virtually the same (insufficient) efficacy [2, 29]. Since there will be no causal treatment in the near future, we think it is time for psychiatry to reconsider its treatment strategies, which are far too one-sidedly fixated on psychopharmacology and pay far too little attention to alternative options, especially in mild cases where psychotherapy without concurrent medication is still a treatment option. Also, regular exercises and sports can definitely be an effective therapeutic means for a considerable number of cases [30, 31]. General practitioners (GPs) are particularly in demand here.

## Conclusions

The results of this study underlined the fact that polypharmacy regimens are not equally suited for every patient. Specifically, such regimens appeared to have a negative impact on treatment response and to obfuscate the “natural” time course of recovery through a multitude of interfering factors. In terms of efficacy, there were no advantages of polypharmacy over monotherapy. Hence, we did not find any substantial benefits that might outweigh the burden of the severe adverse side effects associated with polypharmacy.

We see the following implications for everyday clinical practice: (1) we have to accept that psychotropic drugs or combinations thereof are insufficiently effective in a larger proportion of patients; (2) polypharmacy does not solve this problem in any way—patients often have no benefits whatsoever, but only the burden of more severe adverse reactions; (3) consequently, all therapeutic options must be carefully considered in each individual case; (4) we have further to accept that psychiatric disorders, as they are manifest through the patients’ clinical picture, are likely the result of etiologically very different pathologies, i.e., psychiatric disorders do not represent disease entities in terms of prognosis and therapy; (5) in light of this, it is principally quite unlikely that all patients will respond equally well to a particular therapy and, consequently, we have to think about alternatives, such as regular exercise and sports [30, 31], or the inflammatory response system as targets for therapeutic intervention [cf. 6, 20, 32–35]; (6) in contrast to everyday clinical practice, monotherapy and psychotherapy without concurrent psychopharmacological medications are by no means obsolete—empirical evidence speaks against starting just every therapeutic intervention in psychiatry with a combination of psychopharmaceuticals; (7) we should treat mild cases differently (e.g., mild depression with a HAM-D17 baseline score below 20) and consider to not use psychopharmaceuticals at all, or to opt for psychotherapy alone.

### Limitations

According to the study design, all new hospital admissions were informed about the objectives of our study and invited to participate in this research project. Specifically, patients were informed that participation is voluntary and that they can cancel their participation at any time without giving reasons and without having any disadvantage. Due to the voluntary nature of our study, the obtained selection of study participants must not necessarily be representative of the targeted population of F2 and F3 patients. For example, the chosen recruitment procedure may have resulted in a bias towards more cooperative patients, or towards milder cases, or in the opposite direction towards more severe cases. By design, we have no way to detect such selection biases.

Although of quite respectable size, our sample was not large enough to derive a sufficiently accurate estimate of the differences “polypharmacy versus monotherapy for acute treatment”. Nonetheless, we think that our findings regarding side effect patterns and overall efficacy of therapeutic interventions provide a sound basis to draw reliable conclusions for everyday clinical practice. Due to the great diversity of treatment regimens, dose effects could not be analyzed.

## Footnotes

1 The MEDIS instrument extends the side effects catalog of the AMDP system [16].

2 The PANSS G scale was chosen to prioritize illness-related disabilities in daily functioning over acute symptomatology.

3 This value was achieved during the first week of treatment, after it had been 3.20 ± 1.99 at the time of entry into study.

4 We used the P scale as response criterion, since only this scale reflects the rapid response of patients to therapeutic interventions. This is in contrast to the N and G scales, where psychopathology is only slightly reduced, if at all, during the study period.
